# Successful transition from Treprostinil to Selexipag in patient with severe pulmonary arterial hypertension

**DOI:** 10.1186/s12890-017-0480-9

**Published:** 2017-10-26

**Authors:** Asuka Furukawa, Yuichi Tamura, Hiroya Iwahori, Masato Goto, Narutaka Ohashi, Teruo Okabe, Akio Kawamura

**Affiliations:** 1 Pulmonary Hypertension Center, International University of Health and Welfare Mita Hospital, 1-4-3, Mita, Minatoku, Tokyo, Japan; 2 Department of Cardiology, International University of Health and Welfare Mita Hospital, Tokyo, Japan

**Keywords:** Pulmonary hypertension, Medical therapy, Combination therapy, Prostanoid

## Abstract

**Background:**

In this report, we describe the first successful case of transition from subcutaneous administration of treprostinil to selexipag in a patient with severe pulmonary arterial hypertension (PAH), by evaluating hemodynamic changes and exercise tolerance.

**Case presentation:**

A 38-year-old female with idiopathic PAH (IPAH) had received initial triple combination therapy (macitentan PO, tadalafil PO, and treprostinil SC) and achieved excellent improvement in hemodynamics. Afterwards, due to the development of side effects from subcutaneous administration, we replaced treprostinil therapy with oral selexipag, resulting in stable hemodynamic parameters and exercise capacities.

**Conclusions:**

We report the first case of successful replacement of treprostinil (20.1 ng/kg/min) with selexipag (1600 μg BID) as a component of triple combination therapy, which provides incentive to perform a larger, prospective exchange study.

## Background

Recent reports have supported the benefits of triple combination therapy, including the use of prostacyclin, in patients with severe pulmonary arterial hypertension (PAH) [1]. However, before the release of selexipag, which shows improved morbidity and mortality [2], we could not obtain potent oral prostacyclin and were forced to use parenteral prostacyclin for controlling patients with severe PAH. Therefore, selexipag is expected to replace parenteral prostacyclin usage, especially in patients receiving low dose prostacyclin. In this report, we describe the first successful case of transition from subcutaneous administration of treprostinil to oral selexipag in a patient with severe PAH, as assessed by evaluating hemodynamic changes and exercise tolerance.

## Case presentation

**Fig. 1:**
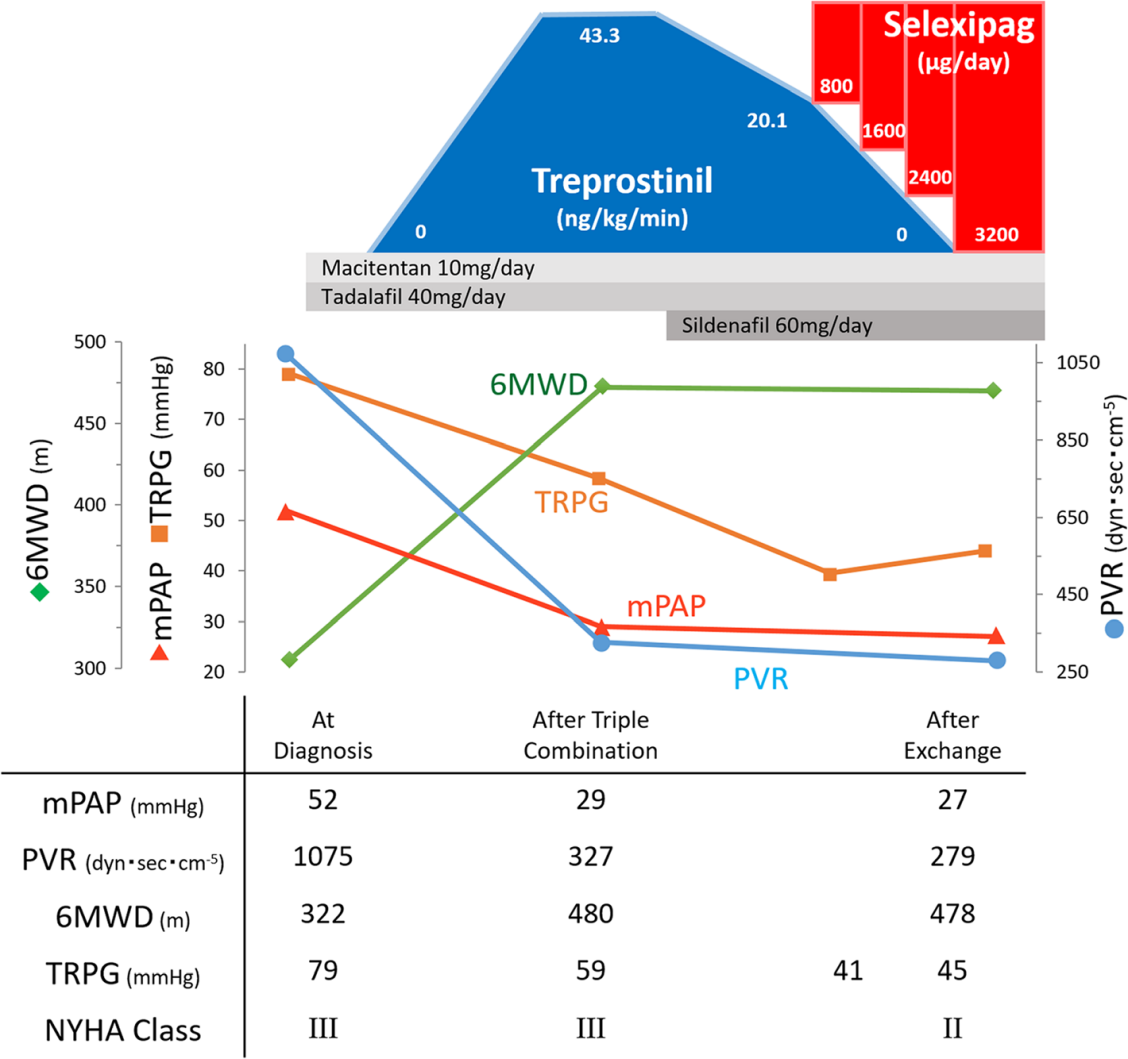
Time course of hemodynamics and exercise capacity. Pulmonary vascular resistance (PVR) and mean pulmonary arterial pressure (mPAP) obtained by right heart catheterization, 6-min walking distance (6MWD), tricuspid regurgitation pressure gradient (TRPG) by echocardiogram, and New York Heart Association (NYHA) class are shown

The patient was a 38-year-old female who was diagnosed with idiopathic PAH (IPAH) 12 months earlier due to the development of dyspnea and peripheral edema. At the initial evaluation, she was classified as New York Heart Association (NYHA) class III with severe symptoms that were associated with high pulmonary arterial pressure (systolic 89, diastolic 30, mean 52 mmHg) and high pulmonary vascular resistance (PVR: 1075 dyn·sec·cm^−5^). She received initial triple combination therapy of macitentan 10 mg PO, tadalafil 40 mg PO, and subcutaneous infusion of treprostinil (43.3 ng/kg/min). Six months after the initiation of treatment, her hemodynamics greatly improved (mean pulmonary arterial pressure 52 to 29 mmHg, and PVR 1075 to 327 dyn·sec·cm^−5^). However, due to the complications of subcutaneous administration (i.e., pain and dermatitis), she strongly wished to cease subcutaneous infusion therapy, so we tried to replace the treprostinil with an oral drug. Initially, we replaced treprostinil with sildenafil 60 mg, which decreased the dosage of treprostinil to 20.1 ng/kg/min. Finally, we exchanged the residual treprostinil with selexipag during a 7-day procedure under careful echocardiographic observation. The patient received an upward titration of selexipag from 400 μg BID to 1600 μg BID within a week, and, in parallel, treprostinil was tapered off (Fig. 1). During the procedure, the patient had no adverse events, such as headache, dyspnea, or hypotension. After completion of the drug exchange (11 months after the initial diagnosis), we performed follow-up evaluations using right heart catheterization and a 6-min walking test, which revealed that the improvements in hemodynamics and exercise capacities had been maintained on the new drug combination (mean pulmonary arterial pressure 29 to 27 mmHg, PVR 327 to 279 dyn·sec·cm^−5^, and 6-min walking distance 480 to 478 m). Furthermore, the improved exercise tolerance was stable 5 months after the exchange.

## Discussion and conclusions

We have reported the first case of successful replacement of subcutaneously infused treprostinil (20.1 ng/kg/min) with oral selexipag (1600 μg BID) with the coadministration of two other drug classes, an endothelin receptor antagonist and phosphodiesterase 5 inhibitors. A recent study revealed that MRE-269, the active metabolite of selexipag, showed similar strong vasorelaxant effects in rat and human pulmonary arteries regardless of the presence of endothelium, which was different from that of treprostinil [3]. This observation suggests that selexipag would perform better than other prostacyclin analogs, especially in severe PAH patients whose pulmonary artery endothelium is damaged and dysfunctional. This study also reported no significant difference in intracellular cyclic adenosine monophosphate levels in human pulmonary artery smooth muscle cells with the same concentrations of MRE-269 and treprostinil (between 10 and 100 nM) [3]. The pharmacokinetics of the drugs also appears to be similar [4, 5]. Moreover, a direct comparison between the active metabolite of selexipag, MRE-269, and other IP receptor agonists by evaluating the vasodilation of rat extralobar pulmonary arteries ex vivo revealed little difference at the same concentrations [3]. These findings suggest that 1600 μg BID oral administration of selexipag is expected to bring at least the same vasodilating effect as 20.1 ng/kg/min subcutaneous infusion of treprostinil does. Furthermore, conventional agents that target the prostacyclin pathway, the prostacyclin analogs, have a disadvantage of a short half-life, while selexipag’s half-life is longer. These findings support that selexipag is a potent substitute for treprostinil or other prostacyclin analogs. To confirm the advantages of selexipag, we need further studies of transitioning from prostacyclin analogs to selexipag. For example, a prospective study is ongoing to evaluate the safety and efficacy of transitions from inhaled treprostinil to oral selexipag [6].

In our case, we also used therapy with dual phosphodiesterase 5 inhibitors. This strategy of using a second phosphodiesterase 5 inhibitor has already been reported to achieve significant improvement in PAH, suggesting that dual phosphodiesterase 5 inhibitor therapy will work well as a salvage therapy in patients with severe PAH. A possible explanation for the added effect of two phosphodiesterase 5 inhibitors on vasodilation in the lungs is that the single approved doses of sildenafil (60 mg/day) and tadalafil (40 mg/day) do not fully inhibit phosphodiesterase 5 in the pulmonary vasculature [7]. Furthermore, some reports strongly support dual or triple combination therapies, including phosphodiesterase 5 inhibitors, endothelin receptor antagonists, and prostacyclin analogs [1, 8].

While there is no head-to-head study to compare the dose effectiveness of selexipag and other prostacyclin analogs, this case report encourages conducting a prospective exchange study that examines the switch of subcutaneous or intravenous prostacyclin analogs to oral selexipag.

## Abbreviations

6MWD: 6-min walking distance
IPAH: Idiopathic pulmonary arterial hypertension
mPAP: Mean pulmonary arterial pressure
NYHA: New York Heart Association
PAH: Pulmonary arterial hypertension
PVR: Pulmonary vascular resistance
TRPG: Tricuspid regurgitation pressure gradient
